# Serial horizontal transfer of vitamin-biosynthetic genes enables the establishment of new nutritional symbionts in aphids’ di-symbiotic systems

**DOI:** 10.1038/s41396-019-0533-6

**Published:** 2019-10-17

**Authors:** Alejandro Manzano-Marín, Armelle Coeur d’acier, Anne-Laure Clamens, Céline Orvain, Corinne Cruaud, Valérie Barbe, Emmanuelle Jousselin

**Affiliations:** 1 UMR 1062 Centre de Biologie pour la Gestion des Populations, INRA, CIRAD, IRD, Montpellier SupAgro, Univ. Montpellier, Montpellier, France; 2 Institut de Biologie François-Jacob, CEA, Genoscope, Évry Cedex, France

**Keywords:** Comparative genomics, Microbial ecology

## Abstract

Many insects depend on obligate mutualistic bacteria to provide essential nutrients lacking from their diet. Most aphids, whose diet consists of phloem, rely on the bacterial endosymbiont *Buchnera aphidicola* to supply essential amino acids and B vitamins. However, in some aphid species, provision of these nutrients is partitioned between *Buchnera* and a younger bacterial partner, whose identity varies across aphid lineages. Little is known about the origin and the evolutionary stability of these di-symbiotic systems. It is also unclear whether the novel symbionts merely compensate for losses in *Buchnera* or carry new nutritional functions. Using whole-genome endosymbiont sequences of nine *Cinara* aphids that harbour an *Erwinia*-related symbiont to complement *Buchnera*, we show that the *Erwinia* association arose from a single event of symbiont lifestyle shift, from a free-living to an obligate intracellular one. This event resulted in drastic genome reduction, long-term genome stasis, and co-divergence with aphids. Fluorescence in situ hybridisation reveals that *Erwinia* inhabits its own bacteriocytes near *Buchnera*’s. Altogether these results depict a scenario for the establishment of *Erwinia* as an obligate symbiont that mirrors *Buchnera*’s. Additionally, we found that the *Erwinia* vitamin-biosynthetic genes not only compensate for *Buchnera*’s deficiencies, but also provide a new nutritional function; whose genes have been horizontally acquired from a *Sodalis*-related bacterium. A subset of these genes have been subsequently transferred to a new *Hamiltonella* co-obligate symbiont in one specific *Cinara* lineage. These results show that the establishment and dynamics of multi-partner endosymbioses can be mediated by lateral gene transfers between co-ocurring symbionts.

## Introduction

Beneficial microbial symbioses have facilitated important ecological transitions in the evolutonary histories of eukaryotes. Countless arthropod species have made use of the metabolic capabilities of bacteria to colonise otherwise unavailable, nutrient-poor ecological niches. Prominent examples include insects that feed on plant sap. These have established obligate associations with obligate bacterial partners that provide them with essential amino acids and vitamins that are lacking in the phloem of their host-plants [1–4]. These bacterial symbionts are generally sheltered within specialised insect cells called bacteriocytes and transmitted from mother to offspring [5]. Consequently, nutritional bacterial symbionts generally persist throughout the diversification of their hosts over evolutionary time scale. These obligate symbioses can sometimes lead to evolutionary “dead-ends”: the vertical transfer of endosymbionts causes severe population bottlenecks, favouring genetic drift and the fixation of slightly deleterious mutations [6–8], leading to the erosion of the endosymbiont’s genome. This so-called ratchet effect can reduce the metabolic versatility of bacteria and alter symbiotic functions [9], ultimately compromising the insect’s adaptive potential. One possible evolutionary solution is to supplement the ancestral symbiont with a new one. A growing body of evidence shows that, in many plant sap feeding insect species, a new obligate bacterial symbiont co-exists with the original one, often taking on a subset of the functions that the “degenerate” symbiont can no longer fulfil [10–12]. These bacterial partnerships can be rather dynamic: the newly arrived symbiont is often replaced during the diversification of the insect [3, 13, 14]. Although these multi-partner endosymbiotic systems are common, their origin and evolutionary stability are not well understood. Which bacteria can become supplementary partners, how tightly they may be integrated into the symbiosis, the different ways they nutritionally complement the primary symbiont, and their turnover during the evolutionary history of their hosts are unclear. It seems possible that new symbionts may not only provide their host with a way of coping with the degradation of the primary symbiont’s genome, but may also contribute novel metabolic functions to the symbiosis [12, 15].

Obligate nutritional endosymbiosis has been extensively studied in aphids (Hemiptera: Aphididae), a group of about 5000 insect species that feed exclusively on the phloem of their host plants. Aphids have been associated for about 150 million years with *Buchnera* aphidicola (Gammaproteobacteria: Enterobacteriaceae) [16], a bacterium that lives within specialised aphid cells called bacteriocytes [5]. Until recently this bacterium was thought to be the sole provider of essential amino acids and vitamins for their hosts. However recent studies have shown that, in some aphid species, *Buchnera aphidicola* co-exists with another bacterium, that is also essential to the nutrition of the host. Among the best documented cases are those that occur in the subfamily Lachninae, a clade of more than 400 species, where *Buchnera aphidicola* has lost the ability to synthesise two essential B vitamins: biotin (B_7_) and riboflavin (B_2_) [14, 17]. As a consequence, both the aphids and their associated *Buchnera* now rely on an additional endosymbiont. These newcomers generally belong to diverse gammaproteobacterial taxa known to be facultative endosymbionts of aphids: *Sodalis* sp., *Fukatsuia symbiotica*, and *Serratia symbiotica* [14, 18]. There is evidence that these new symbionts have been acquired and replaced several times throughout the diversification of their hosts [14, 18], but the factors that cause this turnover remain to be elucidated. Comparative studies show no correlation between the taxonomic identity of the symbiont and diverse aspects of the aphid’s ecology (i.e. taxonomic identity of the host plant, aphid feeding site on the plant, climatic range of the aphid species) suggesting that these new symbionts do not play a prominent role in the adaptation of aphids to new ecological niches [14]. However, this comparative approach looked at niche diversity at a very broad scale and only at the taxonomic identity of the bacteria. A genome-level analysis of the new symbionts is necessary to tell whether they confer new metabolic capabilities on the insect host. Furthermore, although the study of Meseguer et al. [14] suggested that these newly acquired symbionts can be associated with their hosts over a long evolutionary time scale, this assertion relied only on the presence or absence of the bacteria in the aphid. Phylogenomic analyses are needed to assess how long these new partnerships remain stable through evolutionary time and to elucidate the factors that lead to shifts in symbiotic associations.

In this study, we looked at a group of aphids within the Lachninae subfamily in which the new partner of the nutritional symbiosis is a species of *Erwinia*. These bacteria are primarily plant-associated, and include phytopatogenic species [19]. Prior to the study of Meseguer et al. [14], *Erwinia* had only been found in aphids as a gut associate (in a laboratory strain of the pea aphid), and named *Erwinia aphidicola* [20, 21]. The association of *Erwinia* in *Cinara* is therefore of particular interest because of its possible recent transition from a free-living to an obligate endosymbiotic life-style. Its study represents a unique opportunity to understand the mechanisms by which free-living bacteria establish themselves, become integrated as obligate partners, and persist in multi-partner endosymbioses. Here we use fluorescence in situ hybridisation (FISH) and whole-genome sequence data of the endosymbiotic partners of nine *Erwinia*-associated *Cinara* (Aphididae: Lachninae) species to answer the following questions. Are these obligate symbionts derived from a single acquisition of a free-living *Erwinia* lineage? Have they since been integrated in specialised cells and vertically transmitted with their aphid hosts along with *Buchnera aphidicola*? What metabolic functions do these new symbionts bring to the system? Do *Erwinia* provide novel functions unavailable in *Buchnera*? This would suggest that their acquisition could represent an ecological innovation. Alternatively do they merely compensate *Buchnera*’s deficiencies? This could imply that symbiont dynamics is only a succession of bacteria providing similar benefits to their insect host.

We used phylogenomics to shed light on the history of the integration of *Erwinia* as an obligate symbiont and test whether it has been vertically transmitted alongside with *Buchnera*. We then reconstructed the genome-based metabolic complementation of the *Buchnera*-*Erwinia* pairs and explored whether *Erwinia* can confer new metabolic functions to their aphid hosts. These approaches revealed that *Erwinia* endosymbionts have evolved from a single event of lifestyle shift and have then co-diverged with aphids and *Buchnera*. Our data also revealed the existence of a third obligate endosymbiont (*Hamiltonella* sp.) in two closely related aphid species. Investigation of endosymbiont metabolic genes confirmed that *Erwinia* can compensate *Buchnera*’s deficiencies in all aphid species but can also potentially enhance the metabolic capabilities of the symbiotic consortia. Finally, we also showed that some of the nutritional genes that are pivotal in the establishment of the endosymbiosis have been horizontally acquired by *Erwinia* and subsequently passed on to *Hamiltonella*. This suggests that the succession of symbionts in multi-partner symbioses might be both the product of adaptation to new metabolic requirements and the result of displacements of bacteria with similar functions which are acquired through horizontal gene transfer (HGT).

## Materials and methods

### Aphid collection, DNA extraction and sequencing

Following Meseguer et al. [14], we collected nine different species of *Erwinia*-associated *Cinara* aphid species across the north-western and central USA and the south-east of France (supplementary Table S1, Supplementary Material online) and stored in 70% ethanol at 6 °C. For whole-genome sequencing, we prepared DNA samples enriched with bacteria following a modified version of the protocol by Charles and Ishikawa [22] as described in Jousselin et al. [23]. For this filtration protocol 15 aphids from a single colony were pooled together. Extracted DNA was used to prepare two custom paired-end libraries. Briefly, 5 ng of genomic DNA were sonicated, using the E220 Covaris instrument (Covaris, USA). Fragments were end-repaired, 3’-adenylated, and NEXTflex PCR free barcodes adapters (Bioo Scientific, USA) were added by using NEBNext® Ultra II DNA library prep kit for Illumina (New England Biolabs, USA). Ligation products were then purified by Ampure XP (Beckman Coulter, USA) and DNA fragments (>200 bp) were PCR-amplified (2 PCR reactions, 12 cycles), using Illumina adapter-specific primers and NEBNext® Ultra II Q5 Master Mix (NEB). After library profile analysis by Agilent 2100 Bioanalyser (Agilent Technologies, USA) and qPCR quantification, using the KAPA Library Quantification Kit for Illumina Libraries (Kapa Biosystems, USA), the libraries were sequenced, using 251 bp paired-end reads chemistry on a HiSeq2500 Illumina sequencer.

### Fluorescence in situ hybridisation microscopy

From the aforementioned nine species of *Erwinia*-associated *Cinara* species, we investigated symbiont localisation patterns in individuals from *Cinara cuneomaculata*, *Cinara kochiana kochiana* (hereafter referred to as *C. kochiana*), and *Cinara curvipes* in modified Carnoy’s fixative (6 chloroform: 3 absolute ethanol: 1 glacial acetic acid) and left overnight, following the protocol of Koga et al. [24]. Individuals were then dissected in absolute ethanol to extract embryos and transferred into a 6% solution of H_2_O_2_ diluted in absolute ethanol and were then left in this solution for 2 weeks (changing the solution every 3 days). Embryos were then washed twice with absolute ethanol. Hybridization was performed overnight at 28 °C in standard hybridization buffer (20 mM Tris-HCl [pH 8.0], 0.9 M NaCl, 0.01% SDS, and 30% formamide) and then washed (20 mM Tris-HCl [pH 8.0], 5 mM EDTA, 0.1 M NaCl, and 0.01% SDS) before slide preparation. The embryos of up to 10 individuals were viewed under a ZEISS LSM700 confocal microscope. We used two competitive specific probes for *Buchnera* (BLach-FITC [5′-FITC-CCCGTTYGCCGCTCGCCGTCA-FITC-3′], ref. [18]) and *Erwinia* symbionts in *Cinara* (ErCinara-Cy3 [5′-ErCinara-Cy3-CCCGTTCGCCACTCGTCGBCA-3′], this study). TIFF-formatted images were exported using the ZEN v2.3 SP1 software from ZEISS automatically setting the intensity minimum to “black” and the intensity maximum to “white” value (Auto Min/Max option). Exported images were imported into Inkscape v0.92.4 for building the published figures.

### Genome assembly and annotation

Illumina reads were right-tail clipped (minimum quality threshold of 20), using FASTX-Toolkit v0.0.14 (http://hannonlab.cshl.edu/fastx_toolkit/). Reads shorter than 75 base pairs (bp) were dropped. Additionally, PRINSEQ v0.20.4 [25] was used to remove reads containing undefined nucleotides as well as those left without a pair after the filtering and clipping process. The resulting reads were assembled, using SPAdes v3.10.1 [26], with the options --only-assembler option and k-mer sizes of 33, 55, 77, 99 and 127. From the resulting contigs, those that were shorter than 200 bp were dropped. The remaining contigs were binned, using results from a BLASTX [27] search (best hit per contig) against a database consisting of the pea aphid’s proteome and a selection of aphid’s symbiotic bacteria and free-living relatives’ proteomes (supplementary Table S2, Supplementary Material online). When no genome was available for a certain lineage, closely related bacteria were used. The assigned contigs were manually screened using the BLASTX web server (searching against the nr database) to insure correct assignment. No scaffold was found to belong to a *Sodalis*-related bacterium nor contained a 16 SrRNA gene from such bacterial taxa. This binning process confirmed the presence of the previously reported putative *Erwinia* and *Hamiltonella* co-obligate symbionts, as inferred from 16S rRNA gene fragment sequencing from several specimens of the nine *Cinara* species analysed in this study [14, 23], as well as other additional symbionts. For all samples, we identified an additional circular molecule in the *Erwinia* bin representing a putative plasmid. The resulting contigs were then used as reference for read mapping and individual genome assembly using SPAdes, as described above, with read error correction.

The resulting genomes were annotated using Prokka v1.12 [28]. In order to validate start codons, ribosomal binding sites were predicted with RBSfinder [29]. This was followed by non-coding RNA prediction with infernal v1.1.2 [30] (against the Rfam v12.3 database [31]), tRNAscan-SE v1.3.1 [32], and ARAGORN v1.2.36 [33]. This annotation was followed by manual curation of the genes on UGENE v1.28.1 [34] through on-line BLASTX searches of the intergenic regions as well as through BLASTP and DELTA-BLAST [35] searches of the predicted ORFs against NCBI’s nr database. Priority for the BLAST searches was as follows: (1) against *Escherichia coli* K-12 substrain MG1655 (for both *Buchnera* and *Erwinia*), (2) against *Erwinia amylovora* CFBP1430 (for *Erwinia* symbionts) and last (3) against the whole nr database. For each one of these searches, a match was considered valid following manual inspection which took into account identity, domain match, and synteny. The resulting coding sequences (CDSs) were considered to be putatively functional if all essential domains for the function were found, if a literature search supported the truncated version of the protein as functional in a related organism, or if the CDS displayed truncations but retained identifiable domains (details of the literature captured in the annotation files). For *Hamiltonella* symbionts, we performed a draft annotation, using Prokka v1.12, and search and curated over 100 selected genes related to essential amino acids, B vitamins, and other co-factors. For *Erwinia* and *Buchnera* symbionts, pseudogenes were also searched based on synteny against closely related available genomes. This prediction was performed using a combination of sequence alignment with m-coffee [36] and BLASTX searches against the NCBI’s nr database (restricted to *Erwinia* or *Buchnera* taxon ID). This allowed the identification of pseudogenes missed by the previous searches. Metabolic reconstruction for *Buchnera* and *Erwinia* was performed in Pathway Tools v22.5 [37] and manually curated, using EcoCy**c** [38] and BioCyc [39]. Visual plotting of inferred metabolisms was done by hand in Inkscape v0.92.4.

The annotated genomes of *Buchnera* and *Erwinia*, as well as the unannotated ones of *Hamiltonella* and sequencing reads are available at the European Nucleotide Archive with project numbers PRJEB15506, PRJEB31183, PRJEB31187, PRJEB31188, PRJEB31190, PRJEB31191, PRJEB31194, PRJEB31195 and PRJEB31197.

### Phylogenetic analyses

In order to reconstruct the phylogenetic history of the symbiosis and analyse the genetic differences in *Buchnera* and *Erwinia* from the different aphids, we first ran an orthologous protein clustering analysis OrthoMCL v2.0.9 [40, 41] on two sets: (1) *Buchnera* strains from this study + *Buchnera* from *Cinara cedri* as outgroup, and (2) *Erwinia* + *Pantoea* strains + *Erwinia* endosymbionts from this study (supplementary Table S3, Supplementary Material online). We retrieved the single copy-core proteins of the selected genomes per group for phylogenetic reconstruction: 351 protein groups for *Buchnera* and 320 for *Erwinia*. We aligned the single-copy core protein sets, gene by gene, using MAFFT v7.271 [42] (L-INS-i algorithm). Divergent and ambiguously aligned blocks were removed using **Gblocks** v0.91b [43]. The resulting alignments were concatenated for phylogenetic inference. Maximum-likelihood phylogenetic inference were performed in IQ-TREE v1.6.8, using the LG + PMSF + R4 amino acid substitution model and ultrafast bootstrap approximation with 1000 replicates [44–46]. LG was chosen since it incorporates the variability of evolutionary rates across sites in the matrix estimation [47]. The posterior mean site frequency (PMSF) model was chosen as it provides a rapid approximation to the profile mixture models C10 to C60 (variants of the CAT model available in PhyloBayes) [46].

For phylogenetic placement of the *Hamiltonella* symbionts, we used the genes *accD*, *dnaA*, *gyrB*, *hrpA*, *murE*, *ptsI*, and *recJ* following Chevignon et al. [48]. We performed alignments with MUSCLE v3.8.31 [49] and then removed divergent and ambiguously aligned blocks with Gblocks v0.91b. Bayesian inference was performed in MrBayes v3.2.5 [50], using the GTR+I+G substitution model running two independent analyses with four chains each for 3,000,000 generations and checked for convergence.

To infer the origin of the putative horizontally transferred genes, we retrieved orthologous genes across a selection of enterobacterial species (supplementary Table S4, Supplementary Material online). Bayesian inference was performed in MrBayes v3.2.5, using the GTR+I+G substitution model and ran as previously described. Model selection for all nucleotide alignments was done in jModelTest v2.1.10 [51, 52]. Bayesian inference was performed in MrBayes v3.2.5, using the GTR+I+G substitution model and ran as previously described. Model selection for all nucleotide alignments was done in jModelTest v2.1.10 [51, 52].

To infer the origin of the Tn3 mobile elements found in *Erwinia* symbionts’ plasmids, we performed on-line BLASTP vs. NCBI’s nr database and collected the top 50 non-redundant hits. We aligned the sequences with MAFFT v7.271 (L-INS-i algorithm) and then removed divergent and ambiguously aligned blocks woth **Gblocks** v0.91b. Bayesian inference was conducted in MrBayes v3.2.5, using the LG+G substitution model, and ran as described above.

All resulting trees were visualised and exported with FigTree v1.4.1 (http://tree.bio.ed.ac.uk/software/figtree/) and edited in Inkscape v0.92.4. All alignments, NEXUS files, and NEWICK-/NEXUS-formatted trees can be found in 10.5281/zenodo. 2566355.

## Results

### Localisation of *Cinara*-associated *Erwinia* symbionts in aphids

**Fig. 1:**
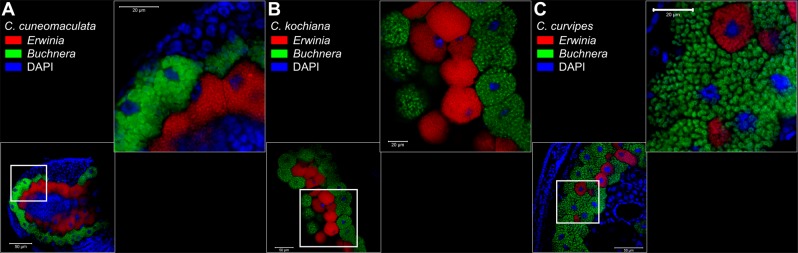
Location and morphology of *Erwinia* and *Buchnera* symbionts in selected *Cinara* aphids. Merged FISH microscopic images of aphid embryos from selected *Cinara* aphids. *Erwinia* signal is shown in red, *Buchnera*’s in green, and DAPI’s (staining DNA, highlighting host nuclei) in blue. Thick white boxes indicate the magnified region depicted in the top-right of each panel. The scientific name for each species along with the false colour code for each fluorescent probe and its target group are shown at the top-left of each panel. Unmerged images can be found in supplementary Fig. S1 (Supplementary Material online)

To investigate the localisation of the *Cinara*-associated *Erwinia* symbionts inside the aphid body, we performed FISH microscopy using probes targeting the 16S rRNA from *Buchnera* and *Erwinia* symbionts of 3 species of aphids: *Cinara cuneomaculata* (Fig. 1a), *Cinara kochiana* (Fig. 1b), and *Cinara curvipes* (Fig. 1c). Similarly to *Buchnera*, the *Erwinia* symbionts were found exclusively distributed inside bacteriocytes distinct from, but in close proximity to, those of *Buchnera*. They present a small coccoid cell shape contrasting with the larger round cells observed for *Buchnera*. This resembles the cellular structure and the localisation of the *Se. symbiotica* symbionts from *Cinara cedri* and *Tuberolachnus salignus* (both holding a highly reduced genome) and contrasts with the tissue tropism of the *Se. symbiotica* symbiont of *Cinara tujafilina* (which holds a mildly reduced genome, displays filamentous cells, and is not confined to its own bacteriocytes) [18].

### Phylogenetic history of the multi-partner endosymbiosis and *Erwinia* genome evolution

We successfully assembled the genomes of nine *Buchnera*-*Erwinia* endosymbiont pairs from *Cinara* aphids. The *Buchnera* assemblies resulted in a single circular chromosome and two plasmids that code for leucine (pLeu, circularised) and tryptophan (pTrp, non-circularised) biosynthetic genes, respectively. The genomes of *Buchnera* are highly conserved in terms of number of genes, and have a size of between 442.57 and 458.25 kilo base pairs with an average G+C content of 23.01%. Average coverages range from 179–6410 × (chromosome), 57–1252 × (pLeu), and 843–9414 × (pTrp) (Supplementary Table S5, Supplementary Material online). These genomes code for an average of 377 proteins, which are largely a subset of those coded by the *Buchnera* strains harboured by *C. cedri*, *C. tujafilina*, and *Cinara strobi*. Regarding the *Erwinia* endosymbionts, we recovered a circular chromosome and a circular plasmid for all strains, with average coverages ranging from 38–402× to 63–1682×, respectively.

**Fig. 2:**
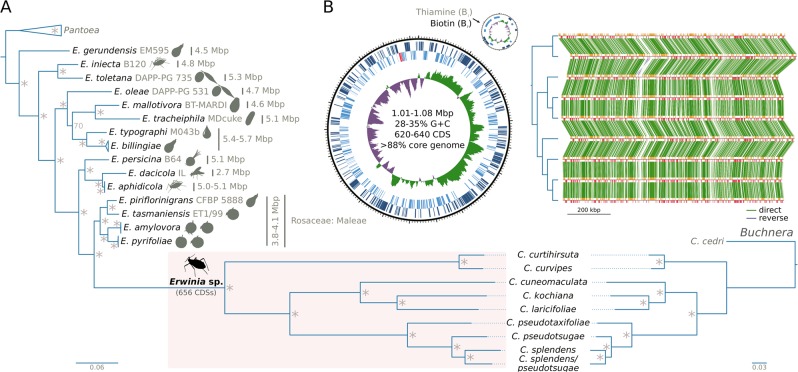
Phylogenetic reconstruction of *Erwinia* spp. and genome properties of *Cinara*-associated *Erwinia* endosymbionts. **a** On the left, phylogenetic reconstruction for *Erwinia* spp., using *Pantoea* spp. as an outgroup. *Erwinia* endosymbionts of *Cinara* species form a well-supported monophyletic group. Silhouettes next to the leaves represents the isolation source for the strains. For the *Erwinia* symbionts, the species name of the *Cinara* host is used. On the bottom right, the phylogeny of the corresponding *Buchnera* strains, using the *Buchnera* associated with *C. cedri* as an outgroup, is shown. Asterisks at nodes of trees stand for a posterior probability equal to 1. **b** On the left, the genome plot for the endosymbiont of *C. pseudotaxifoliae* along with statistics for sequenced *Erwinia* genomes. From outermost to innermost ring, the features on the direct strand, the reverse strand, and GC-skew plot are shown. On the right, pairwise synteny plots of *Erwinia* endosymbionts with a dendogram on the left displaying their phylogenetic relationships

In order to infer the origin and stability of the *Erwinia* symbiont, we built a phylogenetic tree using a set of single-copy core concatenated protein sequences shared across selected *Erwinia* (representing the nine strains recovered here and the currently-available diversity of the genus) and *Pantoea* species (Fig. 2a). A similar approach was adopted for *Buchnera*, we used the nine *Buchnera* genomes recovered here as well as the *Buchnera* associated with *C. cedri* as an outgroup. All *Cinara*-associated *Erwinia* endosymbionts (hereafter referred to as simply *Erwinia* endosymbionts) form a well-supported monophyletic group sister to that of a group of free-living *Erwinia* species that have been isolated as both pathogenic and non-pathogenic Rosaceae plant symbionts. When comparing the *Erwinia* endosymbionts’ tree topology *versus* that of *Buchnera* from the same aphid species, we observed a congruent evolutionary history between the two endosymbionts.

We found that the chromosomes of *Erwinia* endosymbionts all have a very similar genome size of around 1 Mega base pairs (Mbp), an increased A+T content of around 31.50% (when compared to free-living *Erwinia*), an average of 627 CDSs (including the plasmid-encoded proteins) (Fig. 2b; supplementary Table S6, Supplementary Material online), and no mobile elements. Regarding ncRNAs, they all code for a set of 36 tRNAs with charging potential for all of the 20 standard amino acids. All have one tmRNA gene, one 4.5S sRNA component of the Signal Recognition Particle, and the RNase P M1 RNA component. Each genome has an average of 14 pseudogenes, which are largely found as intact CDSs in other *Erwinia* endosymbiont strains. This, in combination with an analysis of shared genes among the *Erwinia* endosymbionts, revealed that the common ancestor of these strains coded for at least 656 distinct CDSs. Regarding genome organisation, the genomes of all sequenced *Erwinia* endosymbionts are highly syntenic (Fig. 2b), with one major inversion of the region delimited by the *dapD* and *fldA* genes.

All *Erwinia* symbionts were found to have a putative multi-copy plasmid, which was recovered in the *Erwinia* bin during the assembly and binning process, regardless of the presence of additional symbionts. This plasmid mainly encodes for proteins involved in the biosynthesis of biotin (*bioA* and *bioB*) and thiamin (*thiC*, *thiF*, *thiS*, *thiG*, *thiH*, *thiD*, and *thiE*) as well as a FAD:protein FMN transferase (*apbE*), a 2,3-bisphosphoglycerate-dependent phosphoglycerate mutase (*gpmA*), a nucleoside permease (*nupC*), a putative heat shock protein (similar to IbpA/IbpB from *Escherichia coli*), and a PLD-like domain-containing protein. The thiamin-biosynthetic genes are notably missing in the plasmids of the *Erwinia* symbionts of *Cinara curtihirsuta* and *C. curvipes*.

### Nutritional complementation of bacterial partners and the tertiary co-obligate *Hamiltonella* symbiont

**Fig. 3:**
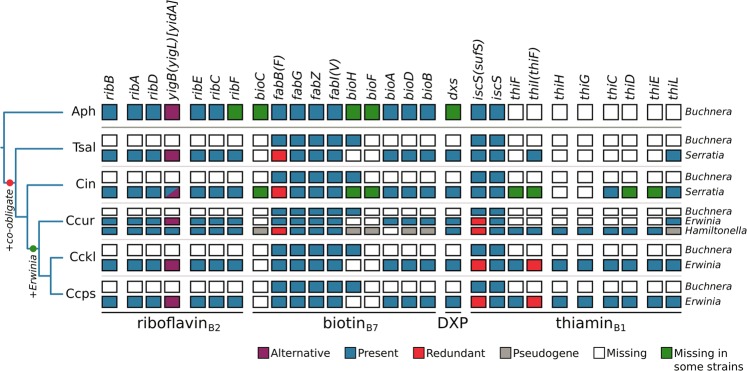
B-vitamin and cofactor biosynthetic metabolic complementation of obligate symbiotic consortia of different aphid species. Diagram summarising the metabolic complementarity of the fixed endosymbiotic consortia of co-obligate symbiotic systems of analysed *Erwinia*-associated *Cinara* aphids. For comparison, a collapsed representation of Aphididae *Buchnera*-only and Lachninae *Buchnera*-*Se. symbiotica* systems are used as outgroups. The names of genes coding for enzymes involved in the biosynthetic pathway are used as column names. Each row’s boxes represent the genes coded by a symbiont’s genome. At the right of each row, the genus for the corresponding symbiont. Abbreviations for the collapsed group of aphids harbouring the symbionts is shown at the left of each group of rows and goes as follows. Aph = Aphididae, Tsal = *T. salignus*, Cct = *C. cedri* + *C. tujafilina*, Ccur = *C. curtihirsuta* + *C. curvipes*, Cckl = *C. cuneomaculata* + *C. kochiana* + *C. laricifoliae*, Cpps = *C. pseudotaxifoliae* + *C. pseudotsugae* + *C. splendens* + *C*. cf. *splendens/pseudotsugae*. On the bottom, lines underlining the genes involved in the pathway leading to the compound specified by the name underneath the line

In previously analysed nutritional di-symbiotic systems in Lachninae aphids, metabolic complementation is observed for some essential nutrients, namely tryptophan, biotin, and riboflavin [14, 17]. *Erwinia* endosymbionts have a highly conserved gene repertoire (supplementary Fig. S2, Supplementary Material online). Unlike their free-living relatives, *Erwinia* symbionts do not retain the capability to *de novo* synthesise any of their host’s essential amino acids (EAAs), but keep specific importers for lysine, arginine, and threonine. Regarding vitamins and cofactors, *Erwinia* symbionts can synthesise riboflavin, biotin, and thiamin pyrophosphate (the biologically active form of thiamin). To infer the dependence of both *Buchnera* and *Erwinia* for nutritional complementation of the aphid, we compared the gene repertoire relating to the biosynthesis of EAAs, vitamins, and co-factors in the nine pairs of genomes from endosymbionts and looked at overlap and complementarity in these nutritional functions (Fig. 3; supplementary Figs. S3 and S4, Supplementary Material online). As stated previously, *Erwinia* have lost most EAAs’ biosynthetic genes, and therefore depend on *Buchnera* for the provision of these nutrients. Similarly to *Se. symbiotica* co-obligate symbionts of Lachninae, the *Erwinia* endosymbionts are able to compensate for *Buchnera*’s gene losses related to the biosynthesis of both riboflavin (full pathway) and biotin (*bioA*, *bioD*, and *bioB* genes). *Erwinia* endosymbionts additionally encode for proteins involved in the biosynthesis of thiamin (*thiF*, *thiI*, *thiH*, *thiG*, *thiC*, *thiD*, *thiE*, and *thiL*), this metabolic function is notably absent from all *Buchnera* genomes as well as all other aphid di-symbiotic systems described so far. While most *Erwinia* preserve intact pathways for thiamin biosynthesis, the symbionts of *C. curtihirsuta* and *C. curvipes* have lost most thiamin-biosynthetic genes.

In both aforementioned *Cinara* species, we actually found a third bacterial partner from the *Hamiltonella* genus [14]. These *Hamiltonella* strains have highly syntenic and reduced genomes of around 1.4 Mbp (supplementary Fig. S5, Supplementary Material online), contrasting the 2.2 Mbp genomes of the *Hamiltonella defensa* facultative symbionts of the pea aphid [48]. Compared to these facultative symbionts, they show little genome rearrangement accompanied by a general loss of both plasmid and phage islands (including the APSE phage). To determine if *Cinara*-associated *Hamiltonella* are distinct *Hamiltonella* species, we calculated Average Nucleotide Identity (ANI) scores [53, 54] *vs*. the facultative *H. defensa* symbiont strain ZA17 (supplementary Table S7, Supplementary Material online), which fell under the recommended species threshold [55]. However, a multi-gene phylogenetic reconstruction shows that the newly sequenced *Hamiltonella* symbionts are nested within the *H. defensa* symbiont clade (supplementary Fig. S6, Supplementary Material online), confidently assigning these symbionts to this taxon. Based on a preliminary genome annotation, both *Cinara*-associated *Hamiltonella* symbionts show a drastically reduced gene repertoire but a similar G+C content when compared with facultative *Hamiltonella* strains (supplementary Table S7, Supplementary Material online), suggesting these newly sequenced *Hamiltonella* symbionts have indeed become obligate. Regarding EAAs, vitamins, and co-factors; *Cinara*-associated *H. defensa* code for the ability to synthesise for L-threonine, lipoic acid, pyridoxal 5’-P (B_6_), riboflavin (B_2_), and most notably thiamin (B_1_) (supplementary Fig. S7, Supplementary Material online). The presence of thiamin-biosynthetic genes perfectly complements the loss of these observed in their partner *Erwinia* endosymbionts (Fig. 3). Compared to the facultative *H. defensa* symbionts, *Cinara*-associated *H. defensa* have lost the ability to synthesise biotin, chorismate, ubiquinol-8, and L-lysine.

### Serial horizontal gene transfer underlies multi-partner co-obligate mutualistic association

**Fig. 4:**
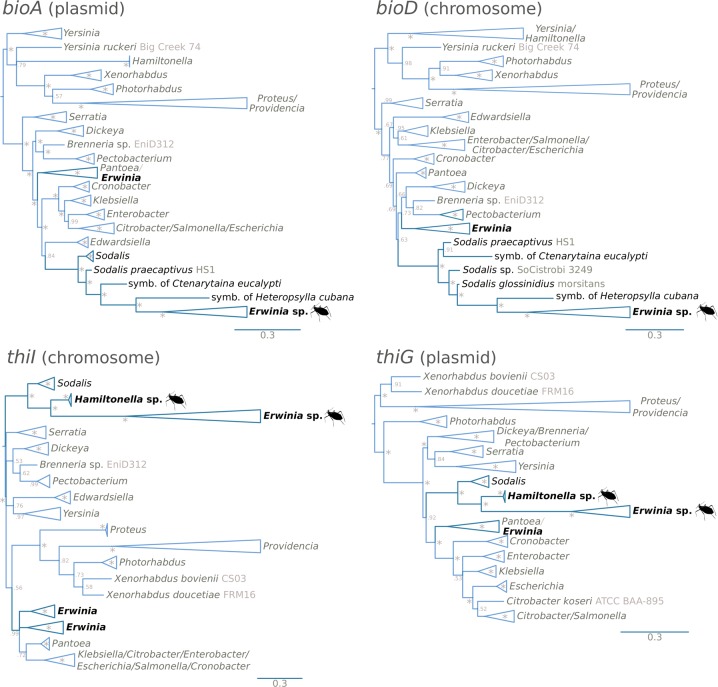
Phylograms of putatively horizontally transferred genes in *Erwinia* and *Hamiltonella* symbionts. Bayesian phylograms for four of the identified putative horizontally transferred genes present in the symbionts of the newly sequenced *Cinara* species. Taxon labels indicate the strain name. Asterisks at nodes stand for a posterior probability equal to 1. On the bottom right, cladogram showing the relationships of genes congruent with the *Erwinia* phylogenetic tree

Both the biotin- and thiamin-biosynthetic genes of the *Erwinia* endosymbiont’s plasmids (*bioA*, *bioB*, *thiC*, *thiE*, *thiF*, *thiS*, *thiG*, *thiH*, and *thiD*) encoded in this molecule consistently showed top BLASTP hits against *Sodalis* and *Sodalis*-like bacteria. Surprisingly, the thiamin-biosynthetic genes present in the *Hamiltonella*’s symbiont genome showed similar results. These results contrasted with what we observed for the plasmid-encoded *nupC*, *apbE*, and *gpmA*, where the top BLASTP hits were consistently against *Erwinia* bacteria. To test for HGT events across the *Erwinia* genome, we ran BLASTP similarity searches of the proteins of *Erwinia* endosymbionts *vs*. a database built from the proteomes of *Erwinia* and *Sodalis* species. The search revealed 11 proteins that putatively originated from an HGT event from *Sodalis*-related bacteria: the plasmidic *bioA*, *bioB*, *thiC*, *thiE*, *thiF*, *thiS*, *thiG*, *thiH*, and *thiD* genes; and the chromosomal *bioD* and *thiI* genes. None of these genes were found to have a “native” copy in the genome that hosts them. To validate these HGT events, we collected orthologous genes across different enterobacterial species and reconstructed Bayesian phylogenies (Fig. 4). All 11 genes supported a single event of HGT for both *Erwinia* and *Hamiltonella* symbionts of *Cinara*, as the *Erwinia* and *Hamiltonella* sequences were consistently recovered as a monophyletic group nested within or sister to *Sodalis* spp. This contrasted with what is observed for the *gpmA* and *nupC* genes, that are confidently recovered nested within *Erwinia* spp. (supplementary Fig. S8, Supplementary Material online). Additionally, the majority of the genes’ subtrees are congruent with the topology of the *Erwinia* endosymbionts’ subtree (supplementary Fig. S9, Supplementary Material online). No *Sodalis*-related bacteria was detected during the assembly and binning process in any of the analysed samples.

**Fig. 5:**
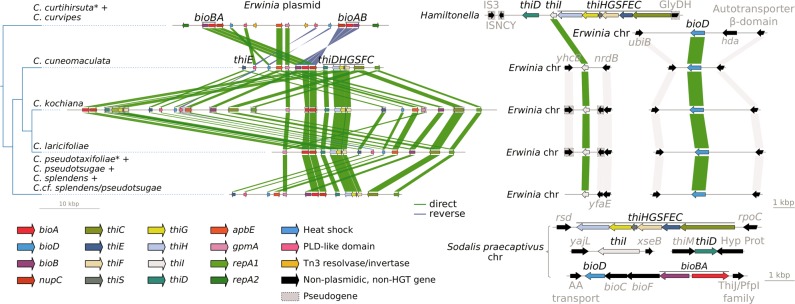
Evolution of the horizontally transferred biotin- and thiamin-biosynthetic genes. Linear plots of the genomic context for the genes originating from a HGT from *Sodalis*-related bacteria. On the left, dendogram showing the phylogenetic relations of the different *Erwinia*-associated *Cinara* groups of species. On the bottom-right, genomic context for the aforementioned genes in *So. praecaptivus* for comparison

To further explore the evolutionary history of the horizontally transferred genes, we analysed their location and gene order in the plasmids of *Erwinia* endosymbionts and the genomes of *Hamiltonella* symbionts and the free-living *Sodalis praecaptivus* (Fig. 5). Although the *Erwinia* endosymbionts’ plasmids are very well conserved in terms of gene content, they display important changes in genome architecture. Within the monophyletic clade made up of the symbionts of *C. kochiana* and *C. laricifoliae*, we observed an ancestral duplication of the full gene repertoire. This event generated a plasmid with one intact and one pseudogenised copy of each gene in *C. kochiana* (except for the heat shock protein) and a new gene order in *C. laricifoliae*. All *Erwinia* endosymbionts have the horizontally transferred *bioD* gene inserted into the same location in the chromosome, suggesting a single event occurred in their common ancestor. Within the monophyletic cluster made of *C. curtihirsuta* and *C. curvipes*, the *Erwinia* endosymbionts show a much different plasmid architecture than those of their sister clade. Their plasmid shows an inverted duplication which includes the *bioA* and *bioB* genes, and notably a Tn3 family resolvase/invertase (a mobile element). The presence of the latter is puzzling, given that the other sequenced *Erwinia* endosymbionts completely lack mobile elements. A Bayesian phylogenetic analysis revealed that this Tn3 family resolvase/invertase is most closely related to Tn3 resolvase/invertases encoded by other *H. defensa* symbionts (Supplementary Fig. S10, Supplementary Material online), hinting at their origin. As mentioned before, the *Erwinia* endosymbionts of *C. curtihirsuta* and *C. curvipes* do not host the typical thiamin-biosynthetic genes in their plasmid. In turn, these genes are now hosted in the *Hamiltonella* genome, flanked by insertion sequence (IS) elements and a glycerol dehydrogenase pseudogenes. The scaffolds in which these genes reside in each *Cinara*-associated *Hamiltonella* strain, and which bear no resemblance to *Erwinia* plasmids, have roughly triple the coverage as the rest of the large scaffolds, suggesting either a triplication in the chromosome or their presence in a multi-copy plasmid (around 1900 vs. 5400× and 620× vs. 1,500×). The plasmidic nature of these molecules is supported by the presence of a replication initiator protein gene and a plasmid segregation protein in the assembled sequences for both *Hamiltonella* strains. In all cases, the *thiL* gene (which is not from HGT origin), coding for the last enzymatic step in the biosynthesis of thiamin diphosphate (the active form in vivo), is located solely in the chromosome of *Erwinia* endosymbionts.

### ‘*Candidatus* Erwinia haradaeae’ sp. nov

We propose the specific name ‘*Candidatus* Erwinia haradaeae’ for the monophyletic lineage of enterobacterial endosymbionts exclusively found affiliated as co-obligate symbionts in the monophyletic group of *Cinara* aphid species analysed in this study, although its presence in other closely related species cannot be discarded. The closest relative of the symbiont of *C. pseudotaxifoliae* by 16S rRNA gene sequence identity is the *Erwinia pyrifoliae* strain EpK1/15 (INSDC accession number CP023567.1) with which it shares 94% sequence identity (2350 bit-score). The specific epithet ‘haradaeae’ is in honour of Hosami Harada, who performed research suggesting the origin of the aphid’s obligate *Buchnera* symbiont as derived from a habitant of the plant on which the host fed [20, 56]. These studies put forward the idea of the aphids’ symbionts possibly evolving from bacteria originally inhabiting the plant, then transitioning to gut associates, and finally obligate symbionts.

In *C. cuneomaculata*, *C. kochiana*, and *C. curvipes*, ‘*Ca*. Erwinia haradaeae’ is found inhabiting the bacteriome inside bacteriocytes distinct from those of *Buchnera*. Previous work referring to these symbionts as ‘*Erwinia*’ or ‘*Erwinia*-related’ include Jousselin et al. [23] and Meseguer et al. [14]. Currently available sequences that correspond to ‘*Ca*. Erwinia haradaeae’ are deposited under INSDC accession numbers LT670851.1 and LT670852.1. All currently known ‘*Ca*. Erwinia haradaeae’ species harbour biotin- and thiamin-biosynthetic horizontally transferred genes from a *Sodalis* or *Sodalis*-related bacteria both in their chromosome and plasmid. The exception to this rule are the strains associated with a co-obligate *Ca*. Hamiltonella defensa symbiont, where the thiamin-biosynthetic genes of horizontal-transfer origin are missing, and in turn are located in a putative plasmid of *Ca*. Hamiltonella defensa. Based on genome-based metabolic inference and their parallel evolutionary history with *Buchnera*, they all are co-obligate endosymbionts along with *Buchnera*, and *Ca*. Hamiltonella defensa in *C. curtihirsuta* and *C. curvipes*, in the *Cinara* species sequenced and presented in this study.

## Discussion

In this work we have investigated the origin and evolutionary stability of a novel multi-partner obligate endosymbiosis between *Buchnera* and *Ca*. Erwinia haradaeae (hereafter *E. haradaeae*) and their aphid hosts.

Our whole-genome-based analyses reveal that the phylogenies of *E. haradaeae* and *Buchnera* are perfectly congruent. This congruency implies that the extant di-symbiotic co-obligate system resulted from a single acquisition of *Erwinia* prior to the divergence of their aphid host. The observed cospeciation pattern also implies that *E. haradaeae* symbionts have then been faithfully vertically transmitted alongside *Buchnera* over evolutionary time scale. This is further supported by the high degree of genome synteny as well as the high similarity in gene content across all analysed strains. The transmission from mother to offspring is further supported by our FISH data on three *Cinara* species. These images show that *E. haradaeae* strains reside in specialised cells (bacteriocytes), close to *Buchnera*, typically observed in vertically transmitted symbionts. Phylogenetic studies of the aphid hosts, the *Cinara* genus, using fossil calibrations, have estimated that the aphid clade hosting *E. haradaeae* is ~20–30 million years old [57, 58]. The di-symbiotic system unravelled here has therefore persisted throughout the Pliocene and Miocene and the diversification of the aphids on their hosts-plants (i.e. mainly *Pseudotsuga*, *Larix*, *Abies*, and a few *Picea* for some species; see see Jousselin et al. [59] and Meseguer et al. [57] for phylogenetic reconstructions of aphid host plant associations in this clade) and across two ecozones (the Palearctic and Nearctic) [57].

Regarding the evolutionary origin of *E. haradaeae*, phylogenetic analyses of *Erwinia* genomes available in GenBank suggest that *E. haradaeae* is most closely related to *Erwinia* species that have been isolated as both pathogenic and non-pathogenic plant-associated bacteria [19]. Other *Erwinia* species have also been found in insects, namely the olive fruit fly (*Bactrocera oleae*) [60], the western flower thrips (*Frankliniella occidentalis*) [61] and aphids [20, 21, 56, 62]. Concerning the latter, *Erwinia* spp. were isolated both from the gut of a laboratory strain of the pea aphid *A. pisum* (i.e. *E. aphidicola*) [20, 21] and from artificial diets exposed to probing by *Diuraphis noxia* (i.e. *Erwinia iniecta*) [62]. The uptake and persistence of *Erwinia* bacteria within the aphid digestive tract is supported by an experiment on *Aphis pomi*. It was shown that this aphid was capable of ingesting a pathogenic *Erwinia amylovora* after a short feeding period and that *Erwinia* would persist in the bodies of the insects for at least 72 h [63]. These studies suggest that the aphid feeding behaviour is a likely source for the presence and persistence of *Erwinia* bacteria inside the aphid digestive tract. This could facilitate a frequent and prevalent interaction between *Erwinia* strains and aphids, which could have preceded the acquisition of *Erwinia* as a symbiotic partner.

The genomes of vertically transmitted symbionts deteriorate over evolutionary time: they lose numerous genes and shrink [9, 64, 65]. This process of genomic erosion has been extensively reported in the literature and seems to unfold rapidly after the transition from a free-living lifestyle to an obligate symbiotic one [66]. *Erwinia* is no exception: the *E. haradaeae* genome is greatly reduced (~1.1 Mbp) in comparison with its free-living relatives (3.83–5.07 Mbp based on currently available genomes), is mostly deprived of mobile elements, and has a reduced gene set relatively rich in house-keeping genes and those involved in their nutritional symbiotic functions. Our analyses also reveal that all nine *Erwinia* genomes reported here are very similar in terms of gene content and gene order. This conserved genomic organization reflects a lack of recombination, possibly resulting from a loss of pathways involved in DNA uptake and recombination [64]. Hence, altogether, our analyses support an evolutionary history involving an early and rapid genome reduction upon transition from a free-living to a host-dependant lifestyle in *E. haradaeae* followed by at least 20 million years of relative genome stasis. This chain of events mirrors the one inferred for *Buchnera* and other obligate endosymbionts [67–69] and suggests that the evolutionary factors driving vertically transmitted obligate symbiont genome shrinkage are common across bacterial lineages.

Regarding the contribution of *E. haradaeae* to the nutrition of its partners, we found that all strains have evolved a compact genome that lost the capacity to synthesise any EAA (supplementary Fig. S2, Supplementary Material online), but that have retained metabolic pathways that are necessary for complementing those of *Buchnera* for the biosynthesis of two B vitamins: biotin and riboflavin. This tightly integrated metabolic complementarity is similar to the one observed in the di-symbiotic partnerships established between *Buchnera and Se. symbiotica* in Lachninae aphids [17] and those found in other plant sap feeders [11, 70–72]. The partitioning of essential nutrient biosynthesis between the two endosymbionts has probably evolved through a three-way coevolutionary process between the aphids and the two bacteria. Though the intricacies of metabolite exchanges in this system remain to be discovered, the separation of *Buchnera* and *E. haradaeae* into distinct bacteriocytes implies that interactions between the two endosymbionts are mediated by the aphid host. In addition, we found that the capacity to synthesise thiamin, another essential B vitamin, was conserved in all newly sequenced symbiotic consortia (Fig. 3). This biosynthetic potential has so far never been found in aphid mono-symbiotic systems (i.e. in *Buchnera* when it is the sole symbiont of the aphid host [69]) nor in the previously analysed nutritional di-symbiotic systems reported in aphids [17]. Regarding the importance of thiamin for the aphids, early experiments showed that the aphid *Neomyzus circumflexus*, a polyphagous species of Aphidinae, had a lower fitness when fed an artificial diet missing thiamin (aphids were smaller and had fewer offspring than when fed a diet including thiamin) [73]. This suggests that thiamin is essential for the aphid development and its reproductive success. The fact that *E. haradaeae*, and *H. defensa* in tri-symbiotic aphids, has retained the set of genes necessary for the synthesis of this vitamin upon its integration as an obligate symbiont of aphids, while other aphid nutritional symbionts studied so far have lost this biosynthetic capacity, suggests that the acquisition of *Erwinia* might compensate for a lower concentration of thiamin in the diet of the aphid hosts. *Cinara* species hosting *Erwinia* are actually the only aphids that feed on *Larix* and *Pseudotsuga* and have radiated on both these host-plant groups [57]. Unfortunately, we cannot tell from these phylogenetic reconstructions whether the acquisitions of these conifer hosts coincide with the acquisition of *Erwinia* and the diversification of the aphid hosts. In order to test the hypothesis that the novel biochemical ability brought by *Erwinia* represents an adaptation to a thiamin-poor phloem, it would be necessary to investigate and compare the vitamin content of the ingested sap of these and other conifer genera.

We found strong evidence that three biotin-biosyntetic genes (*bioA*, *bioD*, and *bioB*) and all thiamin-biosynthetic ones (except for *thiL*) were horizontally transferred once to *E. haradaeae* from a *Sodalis* or *Sodalis*-related bacterium. Our results suggest that these thiamin-biosynthetic genes were subsequently horizontally transferred from the *E. haradaeae* plasmid to the tertiary *Hamiltonella* symbiont (which also shows genomic signatures of long-term obligate symbiosis) in the aphid lineage leading to *C. curtihirsuta* and *C. curvipes*. These were then lost in the associated *E. haradaeae* (Fig. 3). The handing on of the thiamin-biosynthetic genes from *Erwinia* to a new obligate symbiont further supports the hypothesis that these genes are an essential adaptive element of the mutualistic associations between *Erwinia*-associated *Cinara* aphids and their consortium of bacteria. Regarding the putative origin of these transfers, *Sodalis*-allied bacteria are found as facultative or obligate endosymbionts across different insect taxa [12, 72, 74, 75] and likely also in plant tissues [76]. Furthermore, *Sodalis* have been previously found in aphids through 16S rRNA gene sequencing surveys [14, 18, 77]. Therefore, it is likely that *Erwinia* and a *Sodalis*-related facultative endosymbiont could have co-infected aphids, and that such co-infection facilitated the horizontal transfer of these genes to the plasmid of *E. haradaeae* with subsequent transfers of *bioD* and *thiI* to the chromosome. HGT involving genes in vitamin biosynthesis pathways has been previously documented in *Candidatus* Legionella polyplacis, the endosymbiont of the blood-feeding louse *Polyplax serrata* [78]. This *Legionella* symbiont has acquired a complete biotin operon (*bioADCHFB*) likely from a rickettsial bacterium. A similar example is found in the *Cardinium* endosymbionts of the parasitoid wasp *Encarsia pergandiella* [79] and the whitefly *Bemisia tabaci* [78].

**Fig. 6:**
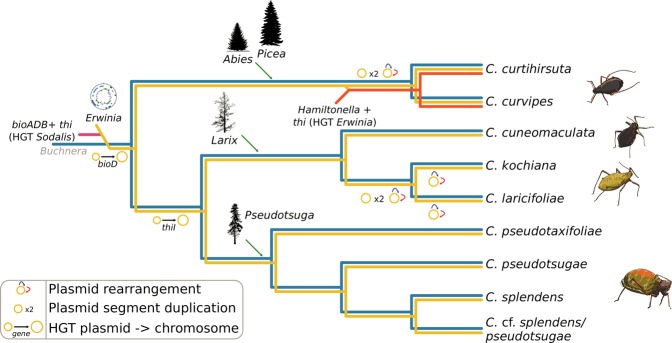
Proposed evolutionary scenario for the establishment and evolution of co-obligate symbionts of *E. haradaeae*-associated *Cinara*. Cladogram displaying the relationships of sequenced *E. haradaeae*-associated *Cinara* lineages. Coloured lines in the dendogram are used to represent the persistence of a symbiont in the aphid lineage. Incoming lines on branches symbolise the acquisition of co-obligate secondary symbionts. Lines joining two symbiotic lineages symbolise HGT events. At the leaves, cartoons of selected aphids form the different groups of genera are shown. Genera and silhouettes of of plant taxa are placed along the branches according to the most likely history of acquisitions inferred from previous studies

The thiamin genes are located in a large putative multi-copy plasmid in both *E. haradaeae* and *Hamiltonella*. This location is not surprising, as nutrient-related biosynthetic genes are often hosted in such extrachromosomal elements [80–84]. It has been proposed for *Buchnera* that the location of EAA-biosynthetic genes in multi-copy plasmids could favour overexpression of the genes of interest, and represent an adaptation to *Buchnera*’s nutritional role [80]. Altogether, our results allow us to propose an evolutionary scenario for the *Buchnera*-*Erwinia* co-obligate symbiotic consortium in *Cinara* aphids (Fig. 6), where an *Erwinia* symbiont replaced the pre-existing co-obligate symbiont by the horizontal transfer of vitamin-biosynthetic genes. Afterwards, in one lineage of *Cinara*, the thiamin-biosynthetic genes of HGT origin were further transferred to a plasmid in a *Hamiltonella* bacterium and in turn lost from the *E. haradaeae* plasmid, thus locking the tri-symbiotic consortium together.

Symbiont replacement is a phenomenon often observed in multi-partner endosymbioses [3, 12, 15], and in such systems the newcomer is often the one that is repeatedly replaced. Our study demonstrates the crucial role that horizontal transfer of pivotal metabolic genes across co-existing symbionts can have in this dynamic: the convergence of metabolic roles in bacterial co-symbionts in this system is the result of HGT between co-residents. The current work also supports the idea, first explored by Harada et al. [56], that the bacteria present in the aphid’s diet are a likely source of obligate symbiotic bacteria. Finally, this work also raises questions about the role of these novel symbionts in the colonisation of new ecological niches. While acquisitions of bacteria in multi-partner endosymbioses studied so far do not involve the recruitment of new nutritional functions [85], here our results suggest otherwise. Indeed, our genomic enquiry suggests that thiamin biosynthesis genes are pivotal in the establishment of both *E. haradaeae* and then *Hamiltonella*, raising questions about the importance of this nutrient for the insect host and therefore the role of host level selection in the process of symbiont replacement.

## Supplementary information

File S1

File S2
